# Numerical Study on Dendritic Growth During Solidification in Vibration-Assisted GMAW of Q450NQR1 Steel Using a Coupled Temperature–Cellular Automaton Model

**DOI:** 10.3390/ma19204328

**Published:** 2026-10-15

**Authors:** Yachen Wei, Dongyuan Lou, Tianxiang Qi, Yang Wang, Xuemei Li, Rui Fan, Hongyan Wang, Li Bao

**Affiliations:** 1 School of Mechanical and Electronic Engineering, Qiqihar University, Qiqihar 161006, China; 18503470693@163.com (Y.W.); 2024936355@qqhru.edu.cn (D.L.); 15556855195@163.com (T.Q.); fanrui_2325@163.com (R.F.); wanghongyan1993@163.com (H.W.); lwjxs2019@163.com (L.B.); 2 The Engineering Technology Research Center for Precision Manufacturing Equipment and Industrial Perception of Heilongjiang Province, Qiqihar 161006, China; 3 Sufei (Jiangsu) Electronic Technology Co., Ltd., Wuxi 214000, China; sup@supfuse.com

**Keywords:** vibration-assisted GMAW, cellular automaton, dendritic growth, grain refinement Solute, distribution

## Abstract

We developed a multiscale numerical model that couples a macroscopic temperature field with a cellular automaton (CA) model to investigate the evolution of the solidification microstructure in the molten pool of Q450NQR1 weathering steel during low-frequency vibration-assisted gas metal arc welding (GMAW). First, finite element analysis was used to obtain the transient temperature field of the molten pool, and temperature data from a representative cross-section were transferred to the CA computational domain. We then incorporated a vibration model and a dendrite fragmentation mechanism into the CA framework to simulate dendrite nucleation, growth, and microstructural evolution under different vibration frequencies and amplitudes. Welding experiments were also conducted to assess the model’s ability to reproduce the response trends associated with changes in vibration parameters. Without vibration, pronounced solute segregation occurred in the molten pool, and the weld microstructure was dominated by columnar grains and coarse equiaxed grains. After vibration was applied, low-frequency mechanical vibration enhanced dendrite fragmentation and promoted the conversion of fragmented dendrites into stable nuclei, thereby facilitating equiaxed grain formation and grain refinement. At a vibration amplitude of 0.10 mm, increasing the vibration frequency from 0 to 50 Hz reduced the average grain size from 68.5 μm to 35.2 μm, corresponding to a grain refinement rate of 48.6%. At 50 Hz, increasing the vibration amplitude further reduced the grain size and improved solute redistribution. These results indicate that, within the parameter range investigated in this study, low-frequency mechanical vibration refines the weld microstructure by promoting dendrite fragmentation and equiaxed grain formation. It also enhances effective solute diffusion during solidification. The simulated microstructural evolution trends agreed well with the experimental observations. These findings provide a basis for optimizing vibration parameters in vibration-assisted GMAW and for controlling the solidification microstructure of welded joints.

## 1. Introduction

Vibration-assisted gas metal arc welding (V-GMAW) extends conventional gas metal arc welding (GMAW) by imposing controlled mechanical vibration on the workpiece during welding. The applied vibration modifies weld-pool solidification and can thereby alter the resulting microstructure and compositional distribution [1,2]. Mechanical vibration is transmitted through the workpiece to the weld pool, where it introduces periodic disturbances that affect solid–liquid interface stability, dendrite growth, and solute redistribution [3]. Near the solidification front, these disturbances may increase the susceptibility of dendrite arms to instability and fragmentation, providing additional potential sources for equiaxed-grain formation [4]. At the same time, vibration-induced disturbances in the liquid phase may enhance solute transport near the solid–liquid interface and reduce local solute concentration gradients, thereby improving the uniformity of the solidification microstructure [5,6]. V-GMAW therefore involves strongly coupled thermal, transport, and solidification phenomena [7]. Because many of these transient mechanisms are difficult to resolve experimentally, numerical simulation provides an important means of interrogating microstructure formation during V-GMAW.

At the microstructural scale, the main numerical approaches include phase-field (PF), Monte Carlo (MC), and cellular automaton (CA) methods [8]. The CA method is particularly attractive because of its relatively low computational cost, its ability to represent competitive grain growth and complex dendritic morphologies, and its straightforward coupling with macroscopic thermal and transport models [9,10]. By describing grain nucleation, dendrite growth, and grain impingement, CA models can reproduce the spatiotemporal evolution of solidification microstructures [11]. In addition, Through spatial interpolation and data mapping, the transient temperature field of the welding molten pool obtained from the macroscopic numerical model can be transferred to the CA computational domain [12], thereby establishing a link between macroscopic temperature evolution and microscopic solidification microstructure simulation [13]. Compared with CA calculations based on a constant thermal gradient or an idealized thermal history, this approach better represents dendrite growth under the highly transient local thermal conditions of a weld pool and provides a suitable framework for examining the effects of vibration parameters on weld solidification.

Previous studies have shown that mechanical vibration can substantially modify weld solidification microstructures, although the dominant mechanisms depend on vibration frequency and amplitude. Ma et al. [14] reported that vibration altered weld-pool solidification morphology and suppressed columnar-grain growth, but they did not systematically resolve dendritic evolution over different vibration conditions. Zinovieva et al. [15] developed a two-dimensional cellular-automaton/finite-difference model for alloy solidification; however, their framework was not formulated specifically for the complex transient conditions of vibration–assisted welding. Wang et al. [16] coupled a thermal model with a CA model and showed that increasing vibration frequency promoted a columnar-to-equiaxed transition, whereas the quantitative links among low-frequency vibration, dendrite fragmentation, equiaxed-grain formation, and solute transport remain incompletely established. Aritaka et al. [17] used fractal analysis to show that increasing vibration frequency increased the morphological complexity of primary aluminum phases, consistent with a more strongly disturbed solid–liquid interface and greater dendrite fragmentation. Many existing vibration-solidification studies, however, focus on ultrasonic or substantially higher frequencies, for which acoustic streaming and cavitation may become important. Those mechanisms are not directly transferable to the 10–50 Hz mechanical-vibration range considered here. Moreover, studies coupling transient weld-pool temperature fields with two-dimensional CA models for low-frequency V-GMAW remain limited, and the respective roles of frequency and amplitude in dendrite fragmentation, grain refinement, and solute redistribution are not yet fully quantified.

Building on the studies described above, our group previously investigated mechanical vibration-assisted GMAW of Q450NQR1 steel from two perspectives: the post-weld microstructure and mechanical properties, and the macroscopic dynamic behavior of the molten pool. These studies provided a basis for understanding how mechanical vibration affects the welding process and the resulting joint microstructure and properties [18,19]. However, previous work focused mainly on the macroscopic response of the molten pool and the characterization of the post-weld microstructure and properties. The microscopic evolution during solidification, including grain nucleation, competitive dendrite growth, dendrite fragmentation, and solute redistribution under vibration, has not yet been described in sufficient detail.

**Figure 1:**
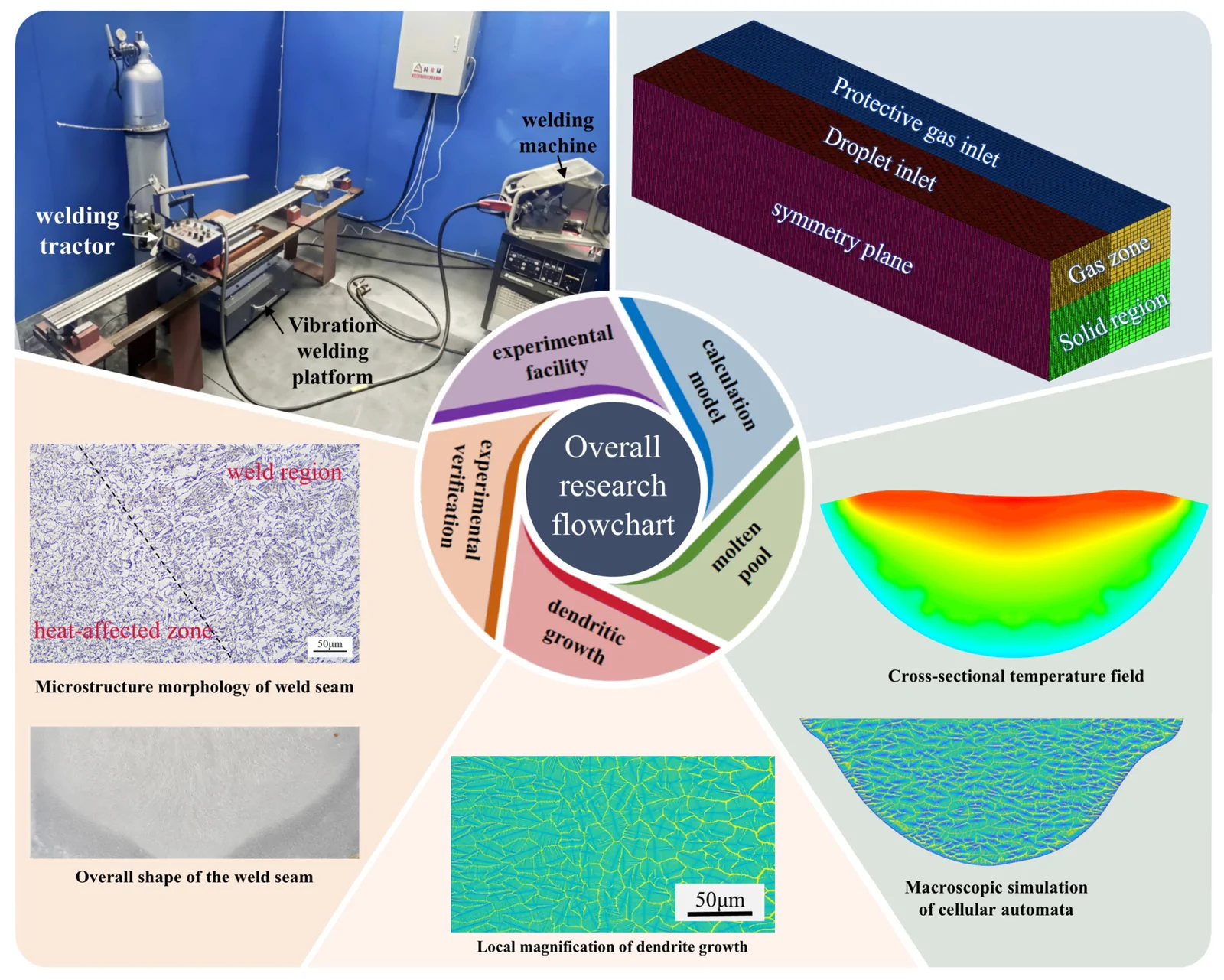
Overall framework for the numerical and experimental investigation of dendritic growth during vibration-assisted GMAW solidification.

In summary, previous studies and our earlier work have revealed the effects of mechanical vibration on molten-pool behavior and the resulting microstructure and properties at different scales. However, the quantitative relationship between low-frequency vibration parameters and dendrite evolution during solidification remains insufficiently understood. Accordingly, this study focuses on Q450NQR1 high-strength weathering steel and develops a two-dimensional CA solidification model driven by a transient macroscopic weld-pool temperature field. Low-frequency vibration effects are introduced through dendrite-fragmentation and effective solute-transport formulations to examine how vibration frequency and amplitude influence dendrite morphology, grain size, and solute redistribution during solidification. V-GMAW experiments performed under corresponding vibration conditions are used to evaluate whether the model captures the observed microstructural trends. The overall research framework is shown in Figure 1.

## 2. Numerical Models and Methods

A two-dimensional solidification model coupling a macroscopic temperature field with a cellular automaton (CA) framework was established to investigate microstructural evolution in a Q450NQR1 steel (Anshan Iron and Steel Group Co., Ltd., Anshan, China) weld pool subjected to low-frequency mechanical vibration. The CA model describes nucleation, competitive dendrite growth, and solute redistribution, while the principal effects of low-frequency vibration are represented through dendrite-fragmentation and effective solute-transport formulations. The vibration range considered in this study is 10–50 Hz. Within this range, the imposed excitation is treated as a periodic mechanical disturbance that affects dendrite loading and liquid-phase solute transport. The model therefore does not explicitly solve the vibration-induced transient Navier–Stokes flow field and does not include Marangoni shear, arc-pressure forcing, droplet transfer, free-surface deformation, or feedback of vibration on the macroscopic temperature field. These simplifications reduce the cost of multiphysics coupling and allow the influence of vibration parameters on dendrite growth and grain refinement to be examined within a tractable temperature-field/CA framework. The material and model parameters are summarized in Table 1.

### 2.1. Solidification Thermodynamics Model

Migration of the solid–liquid interface is driven by thermodynamic undercooling. The present model accounts for thermal, curvature, and constitutional contributions, and the total undercooling is expressed as [20]

(1) $$∆T=T_{L}-T_{X}-\Gamma \cdot K\cdot F+m_{l}\cdot (C_{l}-C_{0})$$

where ΔT is the total undercooling; T_L_ is the liquidus temperature; T_x_ is the local temperature interpolated from the macroscopic thermal field; Γ is the Gibbs-Thomson coefficient; K is the solid–liquid interface curvature; f is the interfacial-energy anisotropy function; ml is the liquidus slope; C_l_ is the liquid-phase solute concentration at the interface; and C_0_ is the initial alloy solute concentration. The term ΓKf represents curvature undercooling, whereas m_l_(C_l_ − C_0_) represents the constitutional contribution associated with solute enrichment.

To account for the effect of crystallographic orientation on dendrite morphology and growth direction, a fourfold anisotropy function is used to describe orientation-dependent interfacial energy [21]

(2) G=1−15ε·cos[4(Φ−θ)]

where ε is the anisotropy-strength parameter, Φ is the angle between the interface normal and the x-axis, and θ is the preferred crystallographic growth direction. This formulation promotes growth along crystallographically favored directions and thus reproduces directional dendritic morphology during solidification.

### 2.2. Temperature-Field Mapping

To couple the macroscopic thermal field to the microstructure calculation, the transient weld-pool temperature field was first obtained using a finite-element model. Temperature data were extracted from the YZ cross-section passing through the initial droplet-impingement position and used as the thermal input to the CA model. The transient macroscopic weld-pool temperature field was obtained using ANSYS Fluent 2024 R2 (ANSYS, Inc., Canonsburg, PA, USA). The two-dimensional cellular automaton (CA) model was implemented in MATLAB R2024b (MathWorks, Natick, MA, USA).

**Figure 2:**
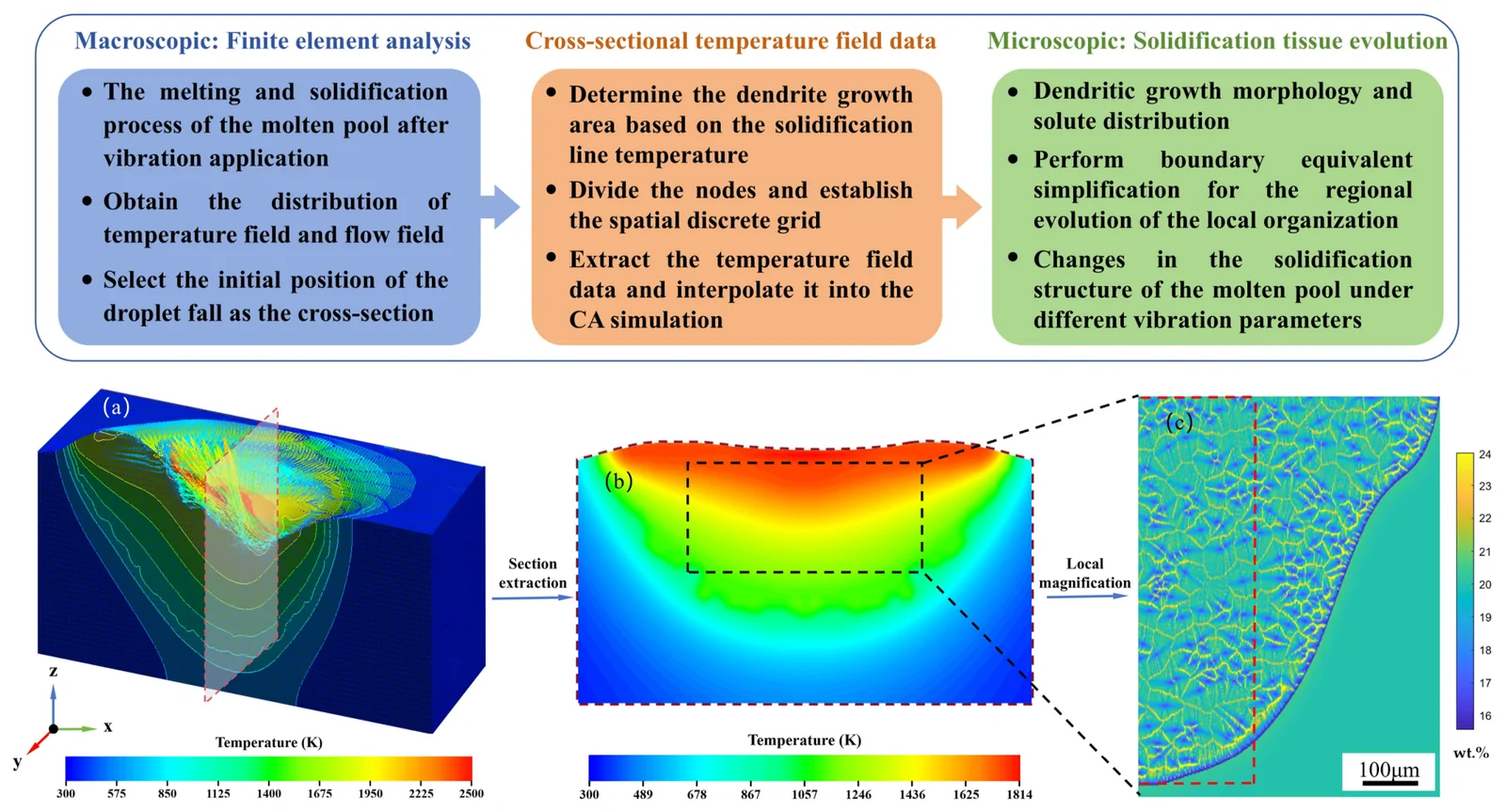
(**a**) Simulation results: Temperature and flow field, (**b**) YZ section temperature field, (**c**) Dendrite growth morphology.

As shown in Figure 2, Figure 2a,b present the macroscopic weld-pool temperature field and the corresponding representative cross-section, whereas the local CA domain is indicated by the red dashed region in Figure 2c. Because the actual weld-pool boundary is curved, direct transfer of the local geometry to the CA grid would complicate boundary treatment and data mapping. The locally curved boundary was therefore approximated by an equivalent straight boundary while preserving, as closely as possible, the temperature gradient and solidification conditions in the region of interest. This approximation provides a regular computational boundary while retaining the dominant local thermal characteristics relevant to dendrite growth.

The macroscopic temperature field was stored as discrete data on a 60 × 15 spatial grid at 15 transient time steps spanning the principal stages of solidification in the region of interest. At each CA time step, the macroscopic temperature field was mapped onto the CA grid using biharmonic spline interpolation [22]

(3) $$f(x,y)=min\iint_{\Omega }{\left(\nabla^{2}f\right)}^{2}dxdy$$

where ∇^2^f denotes the Laplacian operator. This interpolation procedure transfers the time-dependent thermal history from the macroscopic calculation to the CA domain and supplies the temperature field required for subsequent calculations of nucleation, dendrite growth, and solute redistribution.

### 2.3. Nucleation, Growth, and Low-Frequency Vibration

Nucleation during solidification is allowed both at the weld-pool boundary and within the bulk liquid. The number of nucleation sites is calculated as follows [23]

(4) $$N_{s,max}=[n_{s,max}\cdot \pi \cdot r\cdot 10^{-6}]$$

(5) $$N_{v,max}=[n_{v,max}\cdot \left(d_{x}\cdot n_{1}\cdot 10^{-6}\right)\cdot \left(d_{x}\cdot n_{2}\cdot 10^{-6}\right)]$$

where N_s,max_ and N_v,max_ denote the maximum numbers of potential nucleation sites at the weld-pool boundary and in the bulk liquid, respectively; n_s,max_ and n_v,max_ are the corresponding nucleation densities; and r is the weld-pool radius.

The growth-kinetics formulation links the thermodynamic driving force to microstructural evolution by describing advancement of the solid–liquid interface under undercooling, solute redistribution, and the associated evolution of the solid fraction. The interface curvature is evaluated from the discretized solid-fraction field according to [24]:

(6) $$K=\frac{1-2{\bar{f}}_{s}}{d_{x}}$$

Low-frequency mechanical vibration is introduced into the CA framework to represent its influence on solidification-microstructure evolution. The imposed excitation is treated as a periodic mechanical disturbance that affects dendrite loading and liquid-phase solute redistribution; ultrasonic-specific phenomena such as cavitation are not invoked. The vibrating body is assumed to undergo simple harmonic motion, with displacement given by [16]:

(7) $$d(t)=A\cdot sin(2\pi ft+\varphi_{0})$$

Differentiation with respect to time gives

(8) $$v(t)=2\pi \cdot f\cdot A\cdot cos(2\pi ft+\varphi_{0})$$

where d(t) is the instantaneous vibration displacement (μm), v(t) is the instantaneous vibration velocity (μm/s), A is the vibration amplitude, f is the vibration frequency, and φ_0_ is the initial phase.

### 2.4. Dendrite-Fragmentation Model

**Figure 3:**
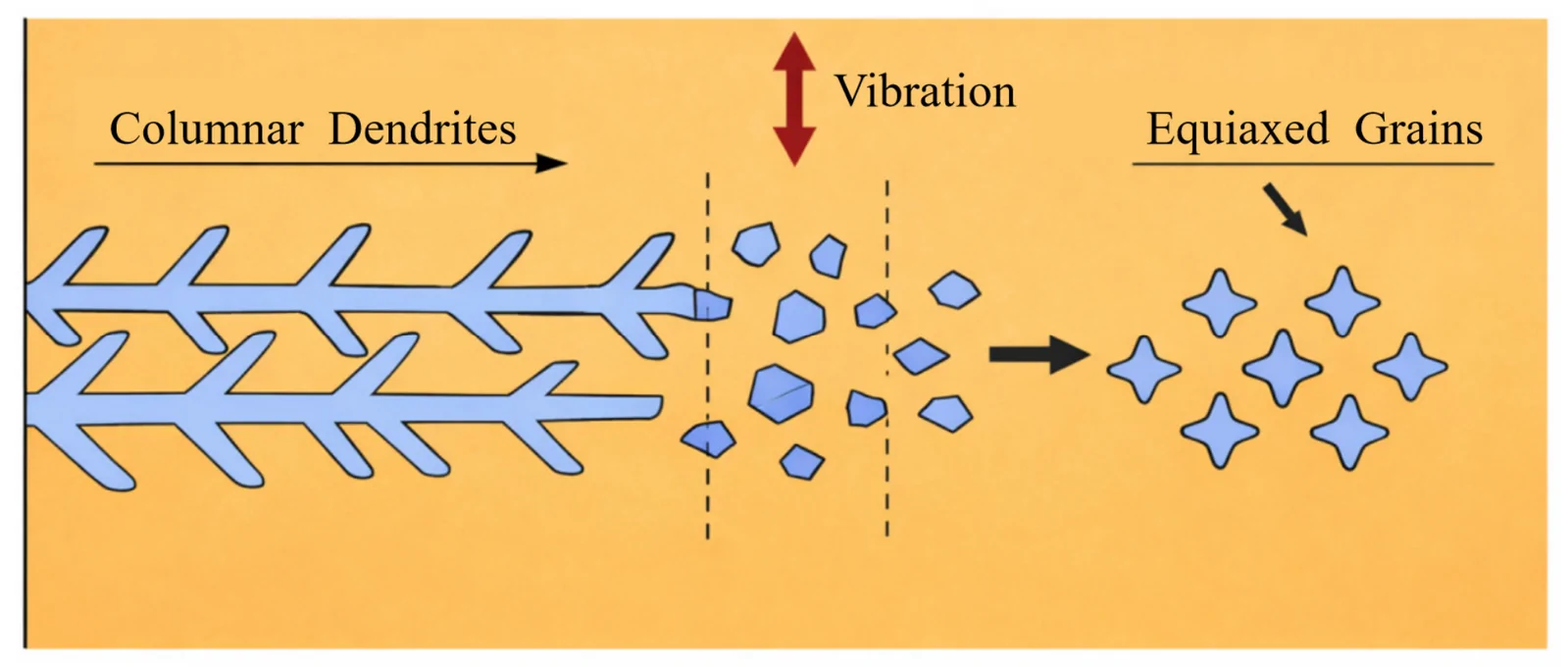
Schematic illustration of vibration-induced dendrite fragmentation.

Under low-frequency mechanical vibration, dendrites near the solidification front experience cyclic mechanical disturbance, and their loading state evolves with the vibration cycle. Dendritic regions with a relatively low solid fraction and weak support from the surrounding solid phase are less mechanically stable and are therefore more susceptible to bending, local instability, and arm fragmentation. Detached fragments that enter sufficiently undercooled liquid may survive and continue to grow if the relevant stability criterion is satisfied, thereby providing additional sites for equiaxed-grain formation. Figure 3 schematically illustrates this fragmentation-mediated nucleation mechanism.

To provide a physical description of vibration-induced dendrite loading, a dendrite arm is idealized as a cantilever beam whose root is connected to the surrounding solid and subjected primarily to periodic inertial loading. The bending stress at the dendrite root is expressed as [25]

(9) $$\sigma_{root}=\frac{F_{v}\cdot R}{W}$$

where F is the equivalent vibration-induced inertial force, R is the dendrite-arm length, and W is the section modulus at the dendrite root (Table 1). This relation indicates that the root bending stress increases with both dendrite length and vibration-induced loading, thereby increasing the tendency for fragmentation.

In the present framework, the cantilever-beam relation is used to establish the physical link between mechanical loading and fragmentation tendency; it is not solved directly within the CA algorithm as a local stress field. Instead, the numerical implementation employs a normalized vibration velocity together with a local dendrite-state function [25]

(10) $$P_{break}=\gamma \cdot \frac{\left|v\left(t\right)\right|}{v_{max}}\cdot H(f_{s})$$

where H(f_s_) is the dendrite-state function, f_s_ is the local solid fraction, and γ is the dendrite fragmentation sensitivity coefficient. Because direct experimental calibration of γ is currently unavailable for the solidification process of Q450NQR1 steel during vibration-assisted GMAW, γ = 0.5 was adopted as the baseline value. A local sensitivity analysis was then performed using γ = 0.3, 0.5, and 0.7. The results show that, within this range, the predicted average grain size varies by no more than approximately 4%, while the overall trend of vibration-induced grain refinement remains unchanged. Therefore, γ = 0.5 was used for subsequent relative comparisons among different vibration conditions rather than being treated as an experimentally calibrated material constant (Table 2).

H(f_s_) is defined as

(11) $$H(f_{s})=\left\{\begin{array}{cc} 1 & if\;f_{s}<0.2\;and\;S_{support}<1.0\\ 0 & otherwise \end{array}\right.$$

where S_support_ is the supporting solid fraction surrounding the dendrite. The state function accounts for both the local solid fraction and the degree of support provided by the surrounding solid. Dendrites with low solid fraction and weak support are assigned a higher fragmentation tendency, whereas increasing solid-phase connectivity during growth improves mechanical stability and reduces the likelihood of breakage. The formulation therefore distinguishes among dendrites at different stages of growth rather than applying an identical vibration effect to all solidifying cells.

Dendrite-fragment survival is governed not only by mechanical loading but also by the local thermodynamic driving force for solidification. The volumetric free-energy difference associated with the liquid-to-solid transformation is expressed as

(12) $$\Delta G_{v}=\frac{L\Delta T}{T_{m}}$$

where L is the latent heat per unit mass (Table 1). Because the volumetric free-energy difference depends on local temperature and solute concentration, it influences the ability of a detached fragment to remain stable and continue growing through its effect on local undercooling.

A fragment generated by low-frequency vibration is not assumed to become a stable CA nucleus immediately. It is first treated as a solid fragment within the surrounding undercooled liquid. Only fragments of sufficient size under favorable local undercooling are allowed to survive and continue growing. A critical nucleation condition is therefore used to determine whether a detached fragment can become a stable new grain [16]

(13) $$r^{*}=\frac{2\lambda_{sl}}{\Delta G_{v}}$$

where r* is the critical nucleation radius and λ_sl_ is the solid–liquid interfacial energy. If the characteristic fragment size satisfies r_frag_ ≥ r*, the fragment is treated as a stable grain nucleus and allowed to grow; otherwise, it is excluded from the population of stable nuclei.

Because the present CA model is two-dimensional, it does not explicitly track the three-dimensional translation, rotation, crystallographic reorientation, or remelting of detached fragments. Fragments satisfying the critical stability criterion are instead represented as new potential nucleation sites. This reduced-order treatment is intended to capture the statistical contribution of fragmentation to equiaxed-grain formation and grain refinement while retaining the computational efficiency of the CA framework.

### 2.5. Solute Redistribution Under Vibration

During solidification, solute partitioning between the solid and liquid phases changes the composition of the interdendritic liquid and thereby modifies constitutional undercooling and dendrite growth at the solid–liquid interface. In the present model, solute partitioning within each CA cell is first described by a local conservation relation [26]

(14) $$C=C_{s}\cdot f_{s}+C_{l}\cdot (1-f_{s})$$

where C is the total solute concentration in a CA cell, Cs is the solid-phase solute concentration, and Cl is the liquid-phase solute concentration. As the solid fraction increases, solute is redistributed between the two phases. When the equilibrium partition coefficient differs from unity, solute enrichment or depletion develops near the advancing interface and alters the local solidification conditions.

Local thermodynamic equilibrium is assumed at the solid–liquid interface. The equilibrium solute concentrations on the liquid and solid sides of the interface are therefore expressed as [26]

(15) $$C_{l}^{*}=C_{0}+\frac{T_{l}-T_{m}+\Gamma KF}{m_{l}}$$

(16) $$C_{s}^{*}=k_{0}C_{l}^{*}$$

where C_l_^*^ and C_s_^*^ are the equilibrium solute concentrations on the liquid and solid sides of the interface, respectively, and k_0_ is the equilibrium partition coefficient. For k_0_ < 1, solute is preferentially rejected into the liquid and accumulates ahead of the solidification front; for k_0_ > 1, solute preferentially partitions to the solid; and for k_0_ = 1, no equilibrium partitioning occurs.

Low-frequency mechanical vibration can induce periodic disturbances in the weld pool and interdendritic liquid, thereby modifying transport across the solute-enriched layer adjacent to the solid–liquid interface [27]. Because the present CA model does not explicitly solve the dendrite-scale velocity field or the full convection-diffusion equation, the additional vibration-induced contribution to solute transport is represented through an effective-diffusion approximation [28]. This reduced-order treatment is intended to capture relative changes in transport strength among vibration conditions, not to resolve local flow direction, vortex topology, or transient convective structures.

To quantify solute segregation under different vibration conditions, a segregation index δ_s_ is introduced

(17) $$\delta_{s}=\delta_{0}\left[1-\chi \frac{A}{A_{0}}\frac{f}{f_{0}}\right]$$

where δ_0_ is the segregation index in the absence of vibration. The reference amplitude A_0_ = 0.10 mm and frequency f_0_ = 50 Hz are used to nondimensionalize the vibration parameters, and χ is the segregation-attenuation coefficient. This empirical relation is used only to describe segregation trends within the low-frequency parameter range examined here.

For liquid-phase solute transport without vibration, the molecular diffusion coefficient Dm is used. Under periodic mechanical vibration, the unresolved disturbance-induced contribution is represented by an effective diffusion coefficient D_eff_ [29]

(18) $$D_{eff}=D_{m}+\frac{A^{2}\omega^{2}}{2}\cdot \tau$$

where ω is the angular frequency and τ is the characteristic time scale associated with the vibration-induced liquid disturbance. Because the dendrite-scale transient flow field is not explicitly resolved, τ is estimated from the characteristic time scale of the imposed periodic excitation:

(19) $$\tau =\frac{1}{\omega }=\frac{1}{2\pi f}$$

The effective-diffusion relation can therefore be rewritten as:

(20) $$D_{eff}=D_{m}+\frac{A^{2}\omega^{2}}{2}\cdot \tau =D_{m}+\pi A^{2}f$$

D_eff_ should not be interpreted as an intrinsic molecular-diffusion coefficient. Rather, it is a reduced-order parameter that represents the additional solute-transport contribution of unresolved liquid-phase disturbances induced by low-frequency mechanical vibration. The approximation is used to compare relative homogenization tendencies among vibration conditions and is not intended to reproduce the local transient convection field.

In addition, the effective diffusion relationship described above is intended to represent unresolved disturbances in the liquid phase. Its purpose is to establish a relative relationship between vibration frequency and amplitude and the solute transport capability in the liquid phase, rather than to replace the complete convection–diffusion equation. Because the transient dendrite-scale velocity field and solute concentration field were not directly resolved in this study, this relationship was not used to predict local convective velocity or transient solute redistribution. Its applicability was instead assessed indirectly by examining the trends in microstructural homogenization under different vibration conditions.

## 3. The Effect of Vibration on Solidification Microstructure

The coupled temperature-field/CA model was used to examine how low-frequency mechanical vibration affects nucleation and dendrite growth, the columnar-to-equiaxed transition, and solute redistribution in the V-GMAW weld pool.

The average grain size is characterized by the two-dimensional equivalent-circle diameter

(21) $$d_{i}=2\sqrt{\frac{S_{i}}{\pi }}$$

(22) $$d_{avg}=\frac{1}{N_{g}}\sum_{i=1}^{N_{g}}d_{i}$$

where d_avg_ is the average grain size, S_i_ and d_i_ are the area and two-dimensional equivalent-circle diameter of the ith grain, respectively, N_g_ is the total number of independent grains in the statistical region, and f_eq_ denotes the equiaxed-grain area fraction.

The equiaxed-grain area fraction is defined as

(23) $$f_{eq}=\frac{S_{eq}}{S_{solid}}\times 100\%$$

where S_eq_ is the total area occupied by equiaxed grains and S_solid_ is the total area of the final solidified region.

### 3.1. Dendrite Growth Evolution

**Figure 4:**
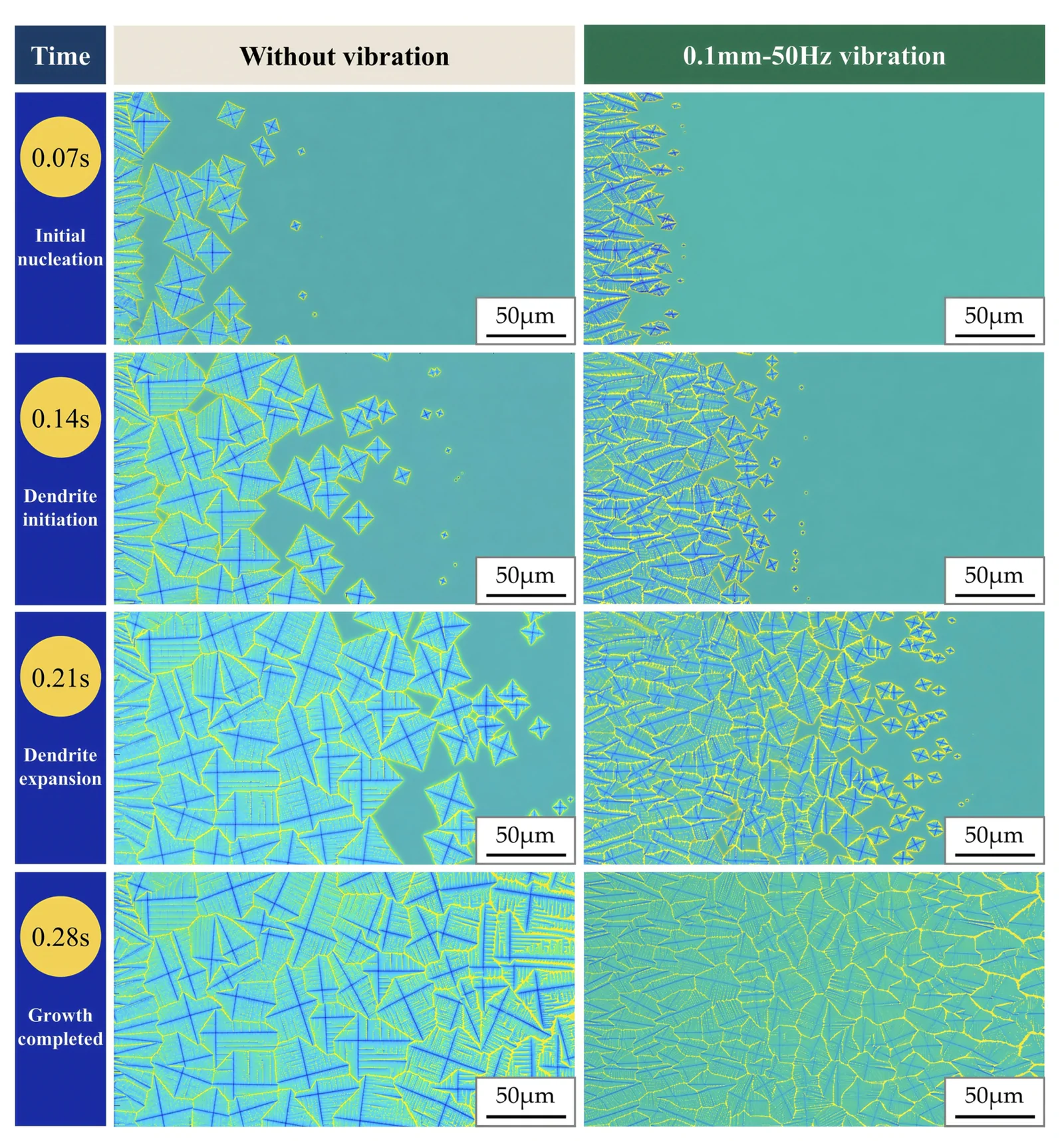
Simulated evolution of the solidification microstructure without vibration and at A = 0.10 mm, f = 50 Hz. The overall grain growth direction is from left to right, consistent with the direction of solidification controlled by the local temperature gradient.

Figure 4 compares the local dendrite-growth sequence without vibration and under low-frequency mechanical vibration at A = 0.10 mm and f = 50 Hz. Identical initial temperature fields, nucleation parameters, and boundary conditions were used in the two calculations so that the modeled effect of vibration on microstructural evolution could be isolated.

**Figure 5:**
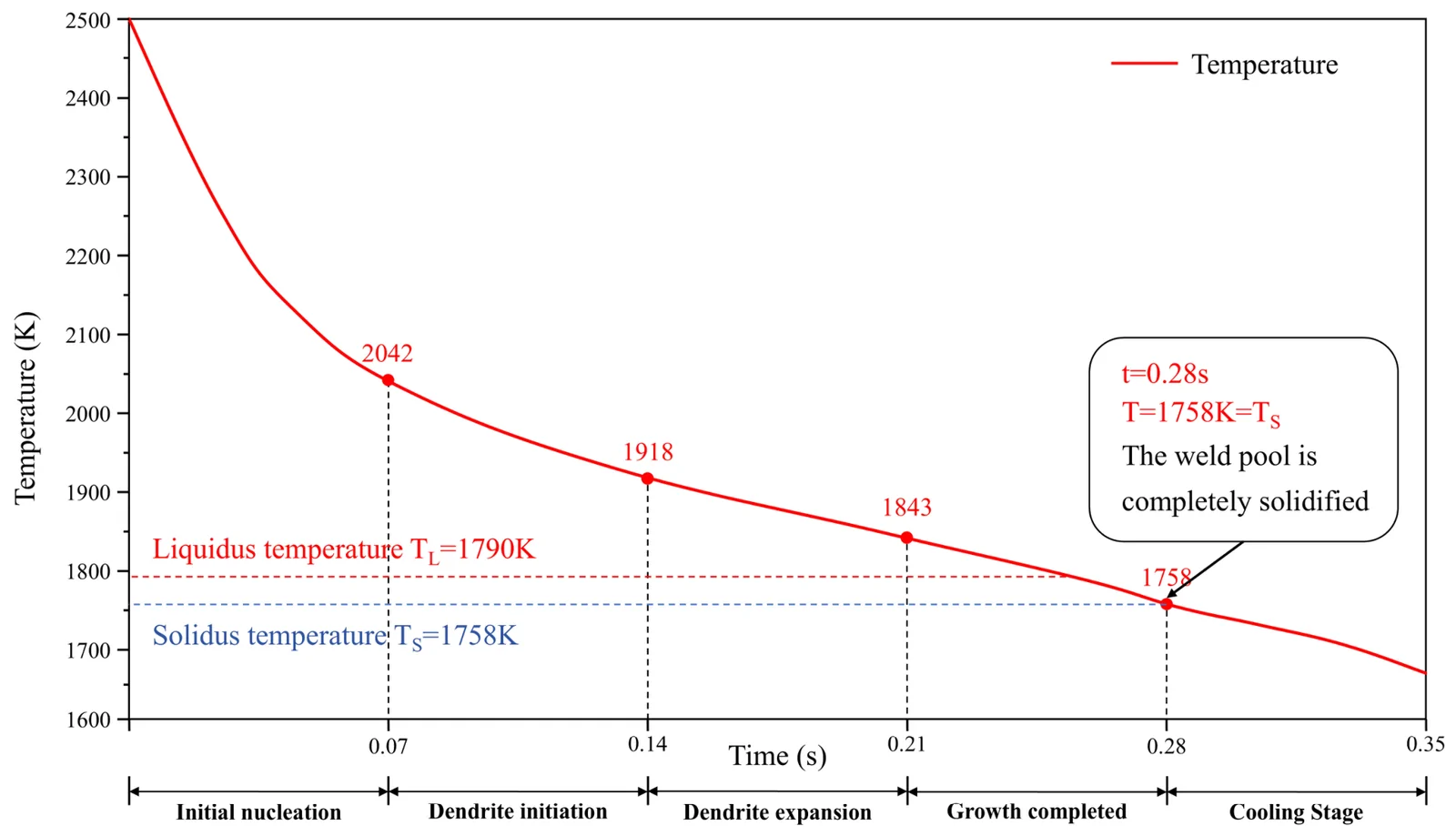
Representative thermal history during weld-pool solidification.

At the onset of solidification, nuclei form near the solid–liquid interface in both cases. With vibration, periodic disturbance of the solidification front produces a denser and more spatially continuous population of nuclei. By t = 0.14 s, dendrites have begun to develop and extend into the liquid. In the non-vibrated case, grains increasingly exhibit preferred growth orientations, whereas the vibrated case contains more fine nuclei ahead of the interface. At t = 0.21 s, dendritic growth accelerates: coarse dendrites in the non-vibrated case continue to extend and impinge on neighboring grains, while vibration-induced fragmentation and fragment-mediated nucleation generate additional equiaxed grains near the solidification front. By t = 0.28 s, solidification is essentially complete in both cases, but the resulting grain morphology and spatial distribution differ markedly. The associated thermal history and solidification stages are shown in Figure 5 [30].

Without vibration, nuclei form primarily near the solid–liquid interface and grow toward the liquid approximately along the local temperature-gradient direction. As solidification proceeds, favorably oriented dendrites gain a competitive advantage, while neighboring dendrites impinge and constrain one another. The resulting microstructure is therefore dominated by columnar grains and relatively coarse equiaxed grains. In the absence of an additional mechanical disturbance, sustained competitive growth of existing grains governs the microstructural evolution.

When vibration is applied at A = 0.10 mm and f = 50 Hz, more independent nuclei appear near the solidification front. At the same time, dendritic regions with low solid fraction and weak surrounding support more readily satisfy the fragmentation criterion used in the model. Fragmentation creates additional potential nucleation sites, which increase the probability of independent grain formation and interrupt the sustained competitive growth of the original columnar grains [31]. As solidification progresses, equiaxed grains occupy a larger fraction of the solidified region, yielding a finer final microstructure with a higher grain number density.

Quantitative analysis shows that the predicted average grain size decreases from 2.72 μm without vibration to 1.94 μm at A = 0.10 mm and f = 50 Hz, corresponding to a reduction of 28.7%. This result confirms that, within the present model, low-frequency mechanical vibration suppresses sustained growth of coarse grains and promotes microstructural refinement.

As shown in Figure 5, the weld-pool temperatures at t = 0.07, 0.14, and 0.21 s are 2042, 1918, and 1843 K, respectively, all above the liquidus temperature TL = 1790 K. These times correspond to the stages of initial nucleation, dendrite initiation, and dendrite extension in the CA calculation. The temperature subsequently decreases to the solidus temperature TS = 1758 K at t = 0.28 s, at which point solidification is essentially complete. The system then enters the post-solidification cooling stage, during which no further substantial evolution of the modeled solidification microstructure is considered.

Overall, the non-vibrated calculation produces a microstructure dominated by columnar grains and coarse equiaxed grains, with pronounced preferred growth and an uneven spatial distribution. Introducing vibration increases the modeled tendency for dendrite-arm fragmentation, and fragment-mediated nucleation provides additional equiaxed grains that interrupt sustained columnar growth. Consequently, the columnar region contracts, the equiaxed-grain fraction increases, and the final structure becomes finer and more uniform. These results are consistent with a vibration-promoted columnar-to-equiaxed transition within the present model.

### 3.2. Effect of Vibration Frequency on Grain Refinement and the Columnar-to-Equiaxed Transition

**Figure 6:**
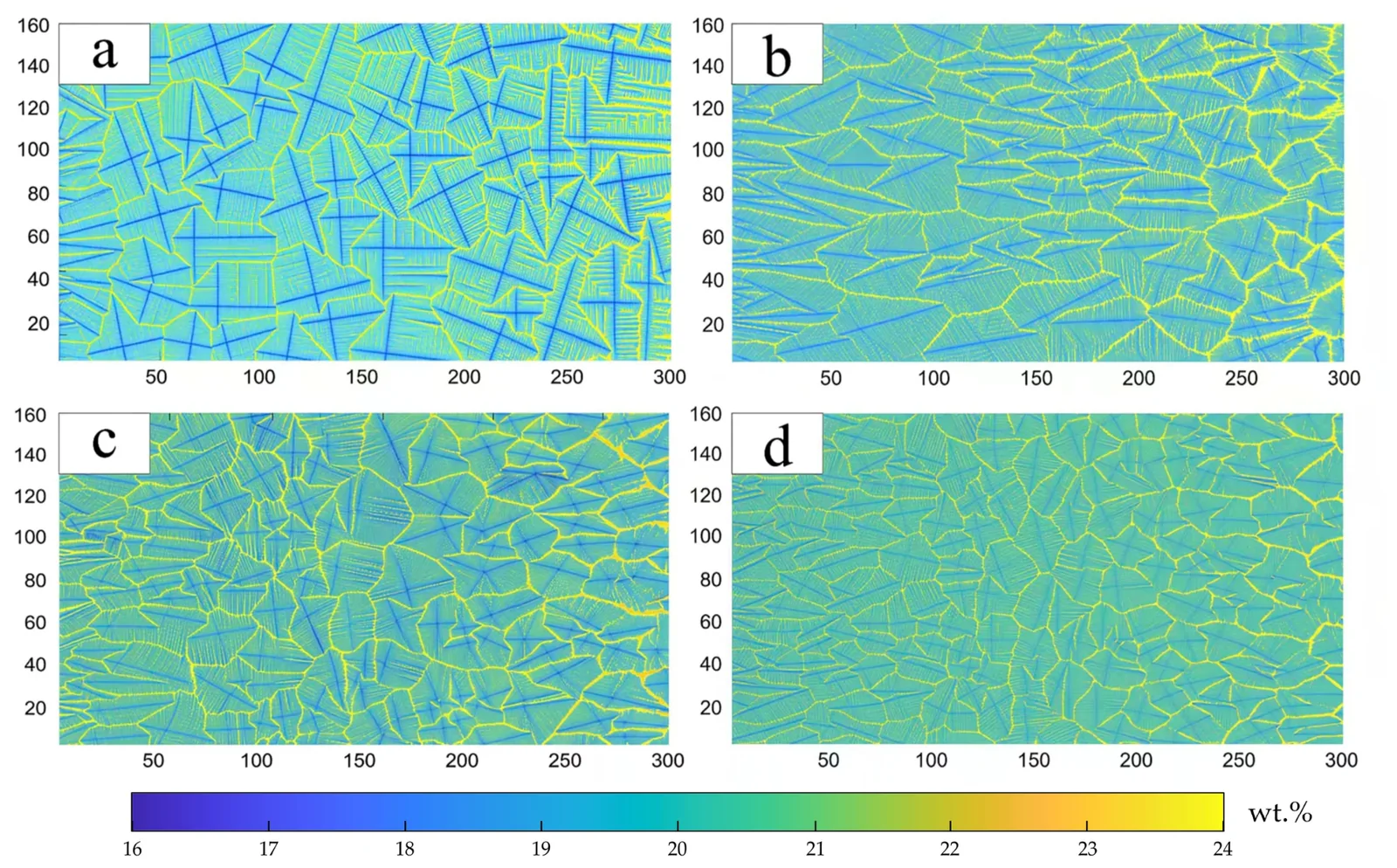
Morphology of dendrite growth at different vibration frequencies under an amplitude of 0.1 mm, (**a**) without vibration, (**b**) 10 Hz, (**c**) 30 Hz, (**d**) 50 Hz. The color bar at the bottom represents the local solute concentration in wt.%. The color transition from blue to yellow indicates an increase in solute concentration from low to high. The coordinate unit is μm.

At a constant amplitude of A = 0.10 mm, vibration frequencies of f = 0, 10, 30, and 50 Hz were compared. As shown in Figure 6, the non-vibrated case is dominated by columnar grains and coarse equiaxed grains, with relatively large, nonuniform grains and pronounced directional growth. At 10 Hz, local side-arm fragmentation appears, fine equiaxed grains begin to form, and the continuity of columnar growth is partially disrupted. At 30 Hz, the columnar region is further reduced, the equiaxed-grain fraction increases, and the structure becomes finer and more uniform. At 50 Hz, the simulated solidified region is dominated by fine equiaxed grains.

### 3.3. Effect of Vibration Amplitude on Solute Redistribution and Microstructural Uniformity

**Figure 7:**
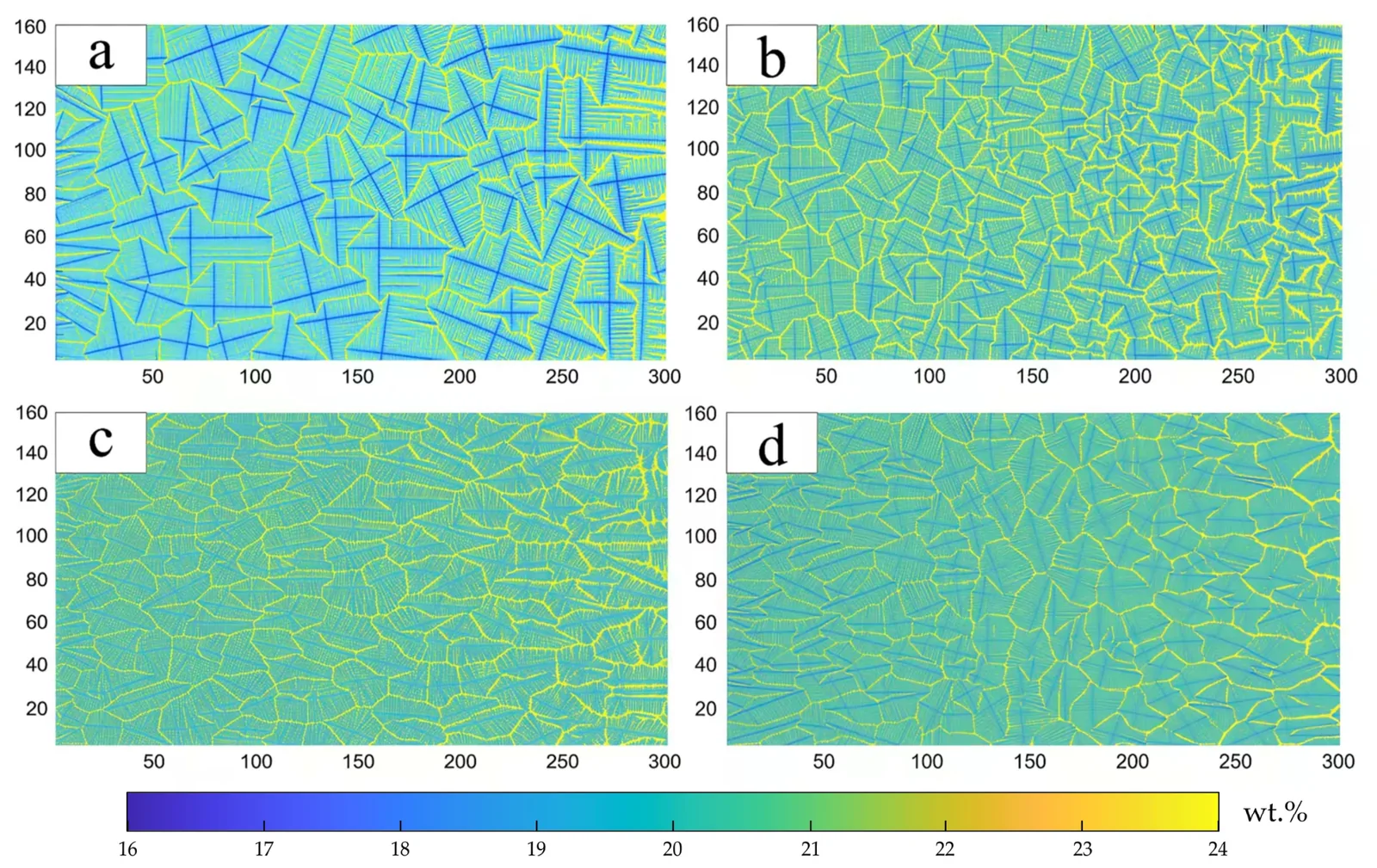
Dendritic growth morphologies under different vibration amplitudes at a constant frequency of 50 Hz: (**a**) non-vibrational condition, (**b**) 0.025 mm, (**c**) 0.075 mm, and (**d**) 0.10 mm.

At a constant vibration frequency of f = 50 Hz, amplitudes of A = 0.025, 0.075, and 0.10 mm were analyzed and compared with the non-vibrated condition. The corresponding simulations are shown in Figure 7 and Table 3.

Without vibration, pronounced interdendritic solute enrichment develops and the simulated microstructure is spatially nonuniform. At A = 0.025 mm, the degree of interdendritic enrichment decreases and the structure becomes more uniform. Further increasing the amplitude to 0.075 and 0.10 mm produces a more homogeneous solute distribution, while the equiaxed-grain fraction increases only modestly. The experimental observations exhibit the same overall tendency, suggesting that vibration amplitude has a stronger influence on homogenization than on the additional reduction in grain size [32].

## 4. Experimental Verification

V-GMAW experiments were conducted at different vibration frequencies and amplitudes to evaluate whether the proposed model captures the experimentally observed response of the weld solidification microstructure to low-frequency mechanical vibration. The weld-center microstructure was characterized quantitatively, and average grain size together with relative grain-refinement rate was compared with the CA predictions reported in Section 3.

The CA model primarily describes grain evolution during solidification, whereas metallographic images reflect the final post-weld microstructure. Therefore, this section focuses on validating the relative grain-refinement trends induced by different vibration parameters rather than directly comparing the absolute grain sizes obtained from the simulations and experiments.

### 4.1. Experimental Materials and Welding Conditions

Q450NQR1 weathering steel (Anshan Iron and Steel Group Co., Ltd., Anshan, China) was used as the base metal and HTW-55 wire (Tianjin Golden Bridge Welding Materials Group Co., Ltd., Tianjin, China) as the filler material. Welding current, voltage, travel speed, and the remaining process conditions were held constant, while either the frequency or amplitude of the applied mechanical vibration was varied. This design ensured comparability among the experimental conditions. The principal material properties and welding parameters are listed in Table 4.

### 4.2. Effect of Vibration Frequency on Weld Microstructure

**Figure 8:**
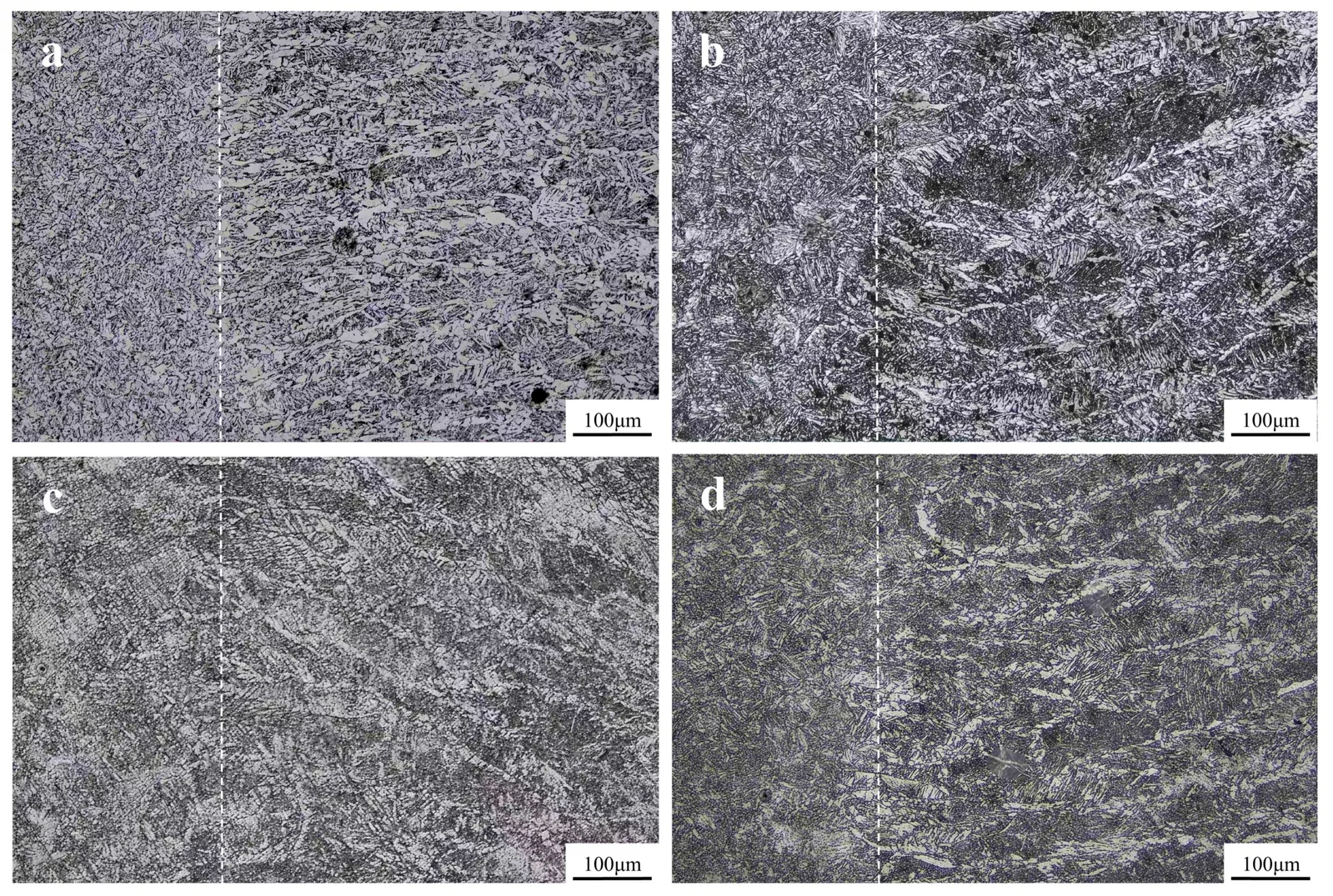
Weld microstructures at different vibration frequencies with a constant vibration amplitude of 0.1 mm: (**a**) 0 Hz; (**b**) 10 Hz; (**c**) 30 Hz; (**d**) 50 Hz. The white dashed lines indicate the boundaries between different microstructural regions of the weld.

To validate the CA model’s ability to reproduce the microstructural trends associated with changes in vibration frequency, four vibration-assisted GMAW experiments were conducted at 0, 10, 30, and 50 Hz while maintaining a constant vibration amplitude of A = 0.10 mm. The microstructure in the weld center was examined, and the grain size was quantified. The weld microstructures obtained at different vibration frequencies are shown in Figure 8. As the vibration frequency increased, the weld microstructure generally exhibited a decrease in coarse columnar structures, an increase in fine equiaxed structures, and improved microstructural uniformity. In addition, the post-weld metallographic microstructure represents the final structure resulting from the combined effects of solidification, subsequent cooling, and solid-state phase transformations. Therefore, the experiments were used primarily to evaluate whether the microstructural trends observed at different vibration frequencies were consistent with the CA simulation results, rather than to provide direct observations of transient dendrite fragmentation during solidification [33,34].

As shown in Figure 8a, under the no-vibration condition, the weld microstructure is dominated by relatively coarse columnar structures and exhibits pronounced directional growth. Relatively continuous ferritic morphologies can be observed near the grain boundaries, while the microstructure is locally nonuniform. These features are consistent with the simulation results, in which sustained competitive growth of columnar grains dominates in the absence of external mechanical vibration.

As shown in Figure 8b, after applying low-frequency mechanical vibration at 10 Hz, the weld microstructure shows a certain degree of refinement. The amount of coarse columnar structure decreases, and more fine grains begin to appear. Compared with the no-vibration condition, the directional growth tendency becomes weaker, although the microstructure remains predominantly columnar. This change is consistent with the CA-model prediction that low-frequency vibration increases the tendency for dendrite fragmentation and creates more potential nucleation sites.

As shown in Figure 8c, when the vibration frequency is increased to 30 Hz, the coarse columnar structure decreases further, while the number of fine grains increases markedly. The overall microstructure also becomes more uniformly distributed. Compared with the 10 Hz condition, the continuity of the columnar structure is further reduced, indicating a more pronounced microstructural refinement response at the higher vibration frequency. This experimental observation is consistent with the CA simulation trend, which shows that increasing the vibration frequency more strongly suppresses sustained competitive dendrite growth and increases the number of potential nucleation sites. However, the metallographic observations alone cannot directly demonstrate the occurrence of transient dendrite-arm fragmentation during solidification.

As shown in Figure 8d, when the vibration frequency is further increased to 50 Hz, the coarse columnar structure decreases substantially, the proportion of fine grains increases further, and the microstructure becomes more uniform. Compared with the lower-frequency conditions, the microstructural morphology at 50 Hz corresponds to the higher dendrite-fragmentation tendency predicted by the CA model. To further quantify the degree of microstructural refinement at different vibration frequencies, the grain size in the weld center was statistically analyzed for each condition. The results are presented in Table 5.

### 4.3. Effect of Vibration Amplitude on the Weld Microstructure

**Figure 9:**
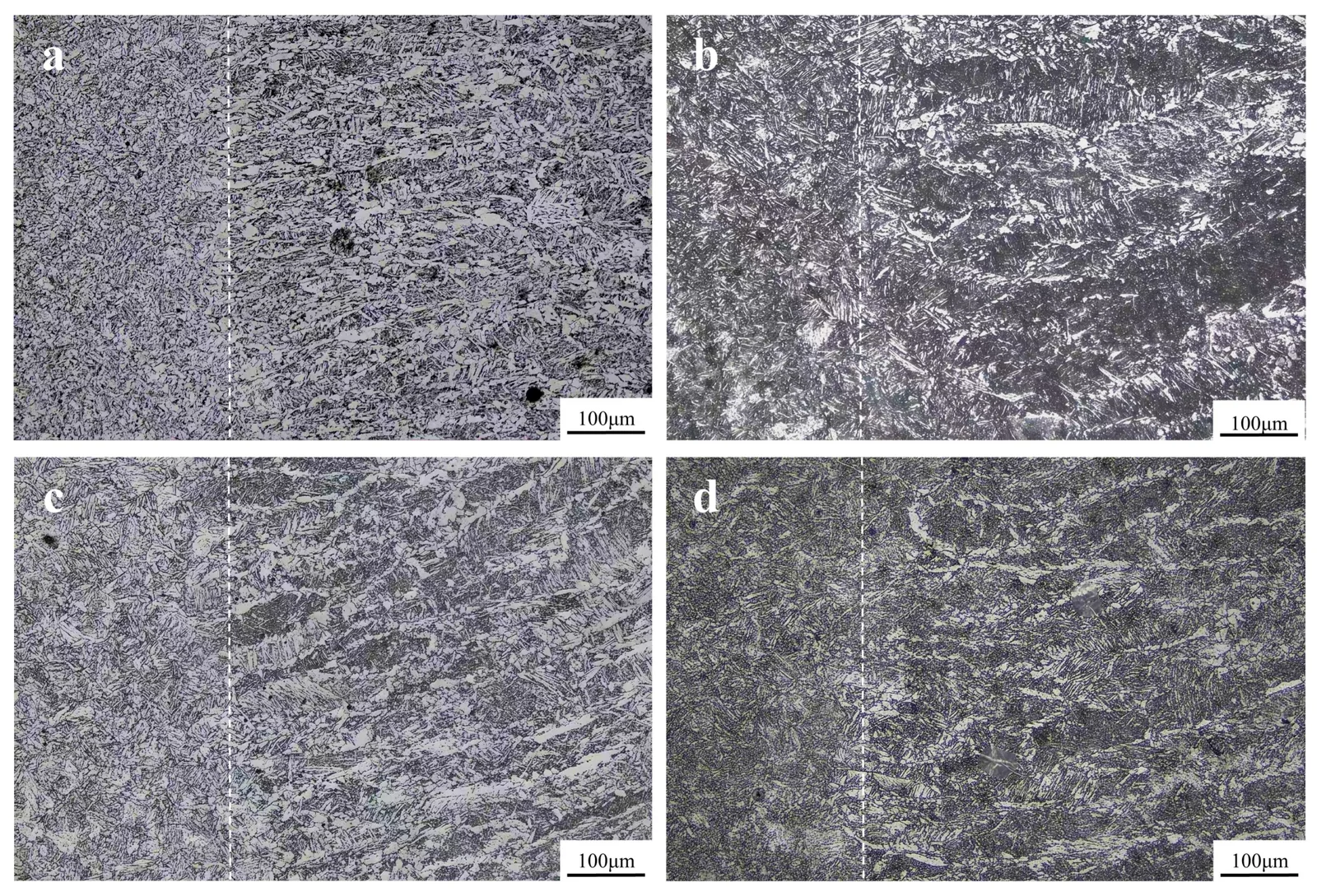
Weld microstructures at different vibration amplitudes with a constant vibration frequency of 50 Hz: (**a**) 0 mm; (**b**) 0.025 mm; (**c**) 0.075 mm; (**d**) 0.10 mm. The white dashed lines indicate the boundaries between different microstructural regions of the weld.

To evaluate the CA model’s ability to capture the microstructural response trends associated with changes in vibration amplitude, four vibration-assisted GMAW experiments were conducted at A = 0, 0.025, 0.075, and 0.10 mm while maintaining a constant vibration frequency of f = 50 Hz. The microstructure in the weld center was examined, and the grain size was statistically analyzed. The weld microstructures obtained at different vibration amplitudes are shown in Figure 9. As the vibration amplitude increased, the weld microstructure generally exhibited a reduction in coarse directionally grown structures, an increase in fine structures, and a progressively more uniform spatial distribution. Because the metallographic observations reflect only the final post-weld microstructure and do not directly measure the transient solute concentration field or liquid-phase flow during solidification, the experimental microstructures were used primarily to evaluate the consistency between the observed trends in microstructural homogenization and grain refinement and those predicted by the CA simulations.

As shown in Figure 9a, under the no-vibration condition, the weld microstructure exhibits a pronounced directional growth tendency. Relatively continuous grain-boundary structures can be observed in some regions, together with a certain degree of spatial nonuniformity. These experimental features correspond to the CA simulation results, in which sustained competitive grain growth and a relatively nonuniform microstructural distribution occur in the absence of vibration.

As shown in Figure 9b, when the vibration amplitude is increased to 0.025 mm, the weld microstructure changes noticeably. The coarse structures decrease, the continuity of the grain-boundary structures is reduced, and the local microstructural distribution becomes more uniform than that under the no-vibration condition. Meanwhile, the average grain size decreases markedly from 68.5 μm to 39.8 μm, indicating that the introduction of a small-amplitude vibration already produces a pronounced grain-refinement effect. This change is generally consistent with the CA-model prediction that vibration promotes grain refinement and improves microstructural uniformity.

When the vibration amplitude is further increased to 0.075 mm, as shown in Figure 9c, the microstructure becomes more homogeneous. The coarse continuous structures decrease further, and the local microstructure becomes more dispersed. The corresponding average grain size is 37.1 μm, which is lower than that at 0.025 mm. However, the reduction is smaller than that observed when the condition changes from no vibration to 0.025 mm. This result indicates that, within the amplitude range investigated in this study, introducing a small vibration amplitude already produces substantial grain refinement, whereas the additional refinement gradually weakens as the amplitude increases further.

As shown in Figure 9d, when the vibration amplitude is increased to 0.10 mm, the weld exhibits the highest degree of refinement and homogenization within the amplitude range investigated. The coarse directionally grown structures are further reduced, and the local microstructural distribution becomes more uniform. At this condition, the experimental average grain size decreases to 35.2 μm, representing a reduction of 48.6% compared with the 68.5 μm obtained under the no-vibration condition. Compared with the 0.025 and 0.075 mm conditions, further increasing the vibration amplitude still reduces the average grain size, but the additional refinement is relatively limited. To further quantify the effect of vibration amplitude on microstructural refinement, the grain sizes obtained under different amplitude conditions were statistically analyzed, as summarized in Table 6.

The experimental results show that the weld grain size exhibits trends similar to those predicted by the CA simulations under different vibration frequencies and amplitudes. However, the CA model provides equivalent grain sizes within a two-dimensional computational domain during solidification, whereas the experimental measurements reflect the final post-weld microstructure. Therefore, the absolute grain sizes obtained from the simulations and experiments are not directly comparable. In addition to reporting the simulated and experimental average grain sizes, this study therefore uses the grain refinement rate relative to the no-vibration condition as a unified metric for comparison. The results are summarized in Table 7.

In addition, Pearson correlation analysis and linear regression were performed using the grain refinement rate. Across all six independent conditions, the Pearson correlation coefficient between the simulated and experimental refinement rates was r = 0.996, with a coefficient of determination of R^2^ = 0.993, indicating a strong linear correlation between the two datasets. Separate analyses of the frequency and amplitude series yielded R^2^ values of 0.992 and 0.998, respectively. This correlation primarily reflects the consistency of the parameter-dependent response trends and does not imply quantitative agreement between the simulations and experiments in terms of absolute grain size or the magnitude of grain refinement.

## 5. Discussion

The simulation and experimental results showed a high degree of consistency in their response to changes in vibration parameters. Correlation analysis based on the grain refinement rate relative to the no-vibration condition gave a Pearson correlation coefficient of r = 0.996 and a linear-regression coefficient of determination of R^2^ = 0.993 across all six independent conditions. Separate analyses of the frequency and amplitude series yielded R^2^ values of 0.992 and 0.998, respectively [35]. These results indicate that the present CA model can reproduce both the direction of the grain-refinement response and the relative ordering of the different vibration conditions. However, the high correlation does not imply quantitative agreement between the simulations and experiments in terms of absolute grain size or the magnitude of grain refinement. Therefore, the present model is more suitable for comparing microstructural response trends among different vibration conditions than for quantitatively predicting the absolute post-weld grain size [36].

For the effect of vibration frequency, both the simulations and experiments showed progressively enhanced grain refinement as the frequency increased. At a fixed vibration amplitude, increasing the vibration frequency increases the number of mechanical disturbance cycles per unit time and raises the characteristic vibration velocity and acceleration. Consequently, the solidification front experiences more frequent periodic mechanical disturbances [36,37]. In the present CA model, this effect increases the fragmentation tendency of dendrites with a low solid fraction and weak surrounding support. It also increases the likelihood that fragments satisfying the stability criterion will act as potential new nucleation sites, thereby suppressing the sustained competitive growth of favorably oriented columnar grains. The frequency range investigated in this study was 0–50 Hz. Within this range, the primary mechanism is associated with low-frequency mechanical disturbance and its influence on solidification microstructure evolution. Therefore, mechanisms dominated by acoustic streaming and cavitation under ultrasonic vibration cannot be directly applied to the present conditions.

The response to vibration amplitude differed from that to vibration frequency. When the amplitude increased from 0 to 0.025 mm, both the simulations and experiments showed pronounced microstructural refinement. Further increases to 0.075 and 0.10 mm continued to reduce the average grain size, but the additional refinement became considerably smaller. At the same time, the experimentally observed continuous grain-boundary structures and locally clustered regions gradually decreased with increasing amplitude, corresponding to the more uniform solute distribution predicted by the simulations. Therefore, the effect of vibration amplitude was reflected not only in grain refinement to some extent, but more clearly in improved microstructural uniformity [32,34]. However, the experiments did not directly measure the transient solute concentration field or liquid-phase flow during solidification. Accordingly, the metallographic observations provide only indirect support for the model-predicted trends of enhanced effective solute diffusion and reduced segregation and should not be regarded as direct validation of the effective-diffusion model.

The overall trends obtained in this study are generally consistent with previous numerical studies of weld solidification. Earlier CA, CA–FE, and phase-field studies have shown that the local thermal history, solid–liquid interface conditions, and solute transport in the liquid phase jointly influence competitive columnar grain growth, equiaxed grain formation, and the resulting solidification microstructure. For example, Chen et al. [9] used a coupled CA–FE model to investigate microstructural evolution during arc-weld solidification and demonstrated significant coupling between the macroscopic thermal process and microscopic grain competition. Wang et al. [11] employed a CA model to investigate dendrite growth and fragmentation under mechanical vibration and reported that mechanical disturbance can increase the likelihood of dendrite fragmentation and subsequent growth of the resulting fragments. Compared with these studies, the present work further incorporates low-frequency mechanical vibration parameters into a transient temperature-field–CA framework for GMAW of Q450NQR1 steel, with particular emphasis on how variations in vibration frequency and amplitude affect dendrite evolution, grain refinement, and solute redistribution.

Compared with our previously published work, which mainly focused on the effects of mechanical vibration on the post-weld microstructure, mechanical properties, and macroscopic dynamic behavior of the molten pool, the present study extends the investigation to microstructural evolution during solidification [18,19]. Particular attention is given to grain nucleation, competitive dendrite growth, dendrite fragmentation, and solute redistribution. The main contribution of this study therefore lies in the coupled temperature-field–CA framework and the numerical description of microstructural evolution at the solidification scale, rather than in a repeated assessment of the effects of mechanical vibration on the final properties of the welded joint.

## 6. Conclusions

A coupled macroscopic transient temperature field–two-dimensional cellular automaton (CA) model was developed to describe the effects of low-frequency mechanical vibration on the solidification microstructure evolution of Q450NQR1 steel during GMAW. Dendrite fragmentation and effective solute diffusion were incorporated to represent the effects of vibration. Within the parameter range investigated in this study, the following conclusions can be drawn.

(1) Low-frequency mechanical vibration alters the competitive grain growth process in the model and increases the tendency for dendrite fragmentation and nucleation from dendrite fragments. As a result, sustained columnar grain growth is suppressed and equiaxed grain formation is promoted. The CA model predicted an average grain size of 2.72 μm under the no-vibration condition. At A = 0.1 mm and f = 50 Hz, the average grain size decreased to 1.94 μm, corresponding to a simulated grain refinement rate of 28.7%.

(2) At A = 0.10 mm, increasing the vibration frequency from 0 to 50 Hz increased the simulated grain refinement rate from 0 to 28.7%. Meanwhile, the experimentally measured average grain size decreased from 68.5 to 35.2 μm, corresponding to an experimental grain refinement rate of 48.6%. Both the simulations and experiments therefore showed progressively enhanced microstructural refinement with increasing vibration frequency.

(3) At f = 50 Hz, increasing the vibration amplitude from 0 to 0.025 mm produced pronounced microstructural refinement. Further increases to 0.075 and 0.10 mm continued to refine the grains, but the additional refinement gradually diminished. Both the simulations and experiments also showed a trend toward improved microstructural uniformity. These results suggest that, within the parameter range investigated, changes in vibration amplitude have a more pronounced effect on effective solute diffusion in the liquid phase and the resulting microstructural uniformity than on further reductions in grain size.

(4) Differences exist between the absolute grain sizes obtained from the simulations and experiments. Therefore, the present model is mainly suitable for analyzing vibration-parameter response trends and the mechanisms of solidification microstructure evolution rather than for quantitatively predicting the absolute post-weld grain size. The main limitations of the current model include its two-dimensional formulation, the simplified treatment of dendrite-fragment motion, the absence of an explicitly resolved vibration-induced transient flow field, and the lack of measurements of the actual local vibration response near the weld. Future work should incorporate three-dimensional flow–solidification coupling, measurements of the actual vibration response, and more comprehensive quantitative microstructural characterization to improve the predictive capability of the model.

## Figures and Tables

**Table 1:** Material and model parameters used in the CA calculations.

Symbol	Parameter Name	Given Value	Unit
T_L_	Liquidus temperature	1790	K
Γ	Gibbs–Thomson coefficient	2.0 × 10^−7^	K·m
m_l_	Liquidus slope	−2.1	K/(wt.%)
C_0_	Initial solute concentration	20	wt.%
ε	Anisotropy strength parameter of interfacial energy	0.05	Dimensionless
r	Melt pool radius	4.0 × 10^−4^	m
n_s,max_	Boundary nucleation density	1.0 × 10^5^	m^−1^
n_v,max_	Internal nucleation density	5.0 × 10^8^	m^−2^
W	Dendrite root cross-section modulus	2.0 × 10^−18^	m^3^
L	Latent heat per unit mass	2.7 × 10^5^	J/kg
k_0_	Partition coefficient	0.7	Dimensionless
λ_sl_	Solid–liquid interfacial energy	0.25	J/m^2^
χ	Segregation weakening coefficient	0.3	Dimensionless
D_m_	Molecular diffusion coefficient	2 × 10^−9^	m^2^/s

**Table 2:** Local sensitivity analysis of γ.

γ	Average Grain Size (μm)	Relative Change Rate
0.3	2.02	4.1%
0.5	1.94	0
0.7	1.87	−3.6%

**Table 3:** Grain size and relative grain-refinement rate predicted by the CA model under different vibration conditions.

Vibration Frequency	Vibration Amplitude	Average Grain Size (μm)	Relative Grain-Refinement Rate
0 Hz	0 mm	2.72	0%
10 Hz	0.10 mm	2.42	11.0%
30 Hz	0.10 mm	2.16	20.6%
50 Hz	0.025 mm	2.08	23.5%
50 Hz	0.075 mm	1.99	26.8%
50 Hz	0.10 mm	1.94	28.7%

**Table 4:** Principal material properties and welding parameters.

Property	Value	Property	Value
Dynamic viscosity	5.0 × 10^−3^ Pa·s	Latent heat of fusion	5.89 × 10^5^ J·kg^−1^
Solidus temperature	1758 K	Liquidus temperature	1790 K
Droplet inlet velocity	0.155 m·s^−1^	Welding current	260 A
Droplet temperature	2500 K	Welding voltage	28 V
Density	7850 kg·m^−3^	Thermal expansion coefficient	1.2 × 10^−5^ K^−1^

**Table 5:** Experimental grain size and relative grain-refinement rate at different vibration frequencies.

Vibration Frequency	Vibration Amplitude	Average Grain Size (μm)	Relative Grain-Refinement Rate
0 Hz	0 mm	68.5	0%
10 Hz	0.10 mm	53.8	21.5%
30 Hz	0.10 mm	42.1	38.5%
50 Hz	0.10 mm	35.2	48.6%

**Table 6:** Experimental grain size and relative grain-refinement rate at different vibration amplitudes.

Vibration Frequency	Vibration Amplitude	Average Grain Size (μm)	Relative Grain-Refinement Rate
0 Hz	0 mm	68.5	0%
50 Hz	0.025 mm	39.8	41.9%
50 Hz	0.075 mm	37.1	45.8%
50 Hz	0.100 mm	35.2	48.6%

**Table 7:** Comparison of Simulated and Experimental Grain Refinement.

Process Parameters	Simulated Grain Size/μm	Experimental Grain Size/μm	Simulated Refinement Rate/%	Experimental Refinement Rate/%	Difference/Percentage Points
0 Hz, 0 mm	2.72	68.5	0	0	0
10 Hz, 0.10 mm	2.42	53.8	11.0	21.5	10.5
30 Hz, 0.10 mm	2.16	42.1	20.6	38.5	17.9
50 Hz, 0.025 mm	2.08	39.8	23.5	41.9	18.4
50 Hz, 0.075 mm	1.99	37.1	26.8	45.8	19.0
50 Hz, 0.10 mm	1.94	35.2	28.7	48.6	19.9

## Data Availability

The original contributions presented in this study are included in the article. Further inquiries can be directed to the corresponding author.
